# Supplementary material for: SARS-CoV-2 virulence factor ORF3a blocks lysosome function by modulating TBC1D5-dependent Rab7 GTPase cycle
Source: Nat Commun. 2024 Mar 6;15:2053. doi: 10.1038/s41467-024-46417-2 (PMC10918171; doi:10.1038/s41467-024-46417-2)
Supplement: Supplementary file 1 — Supplementary Information [file 41467_2024_46417_MOESM1_ESM.pdf]

Supplementary Information

SARS-CoV-2 virulence factor ORF3a blocks lysosome function by modulating TBC1D5-dependent Rab7 GTPase cycle

Kshitiz Walia[1,2], Abhishek Sharma[1], Sankalita Paul[3], Priya Chouhan[1,2], Gaurav Kumar[1], Rajesh Ringe[1], Mahak Sharma[3] and Amit Tuli[1,2*]

Affiliations:

[1]Division of Cell Biology and Immunology, CSIR-Institute of Microbial Technology (IMTECH), Chandigarh, India
[2]Academy of Scientific and Innovative Research (AcSIR), Ghaziabad, Uttar Pradesh, India
[3]Department of Biological Sciences, Indian Institute of Science Education and Research (IISER), Mohali, Punjab, India

[*]Corresponding author

Email: atuli@imtech.res.in

Running title: ORF3a impairs the Rab7 GTPase cycle

Keywords: ORF3a, Rab7, Arl8b, TBC1D5, HOPS, Vps39, Lysosome

SUPPLEMENTARY FIGURE S1

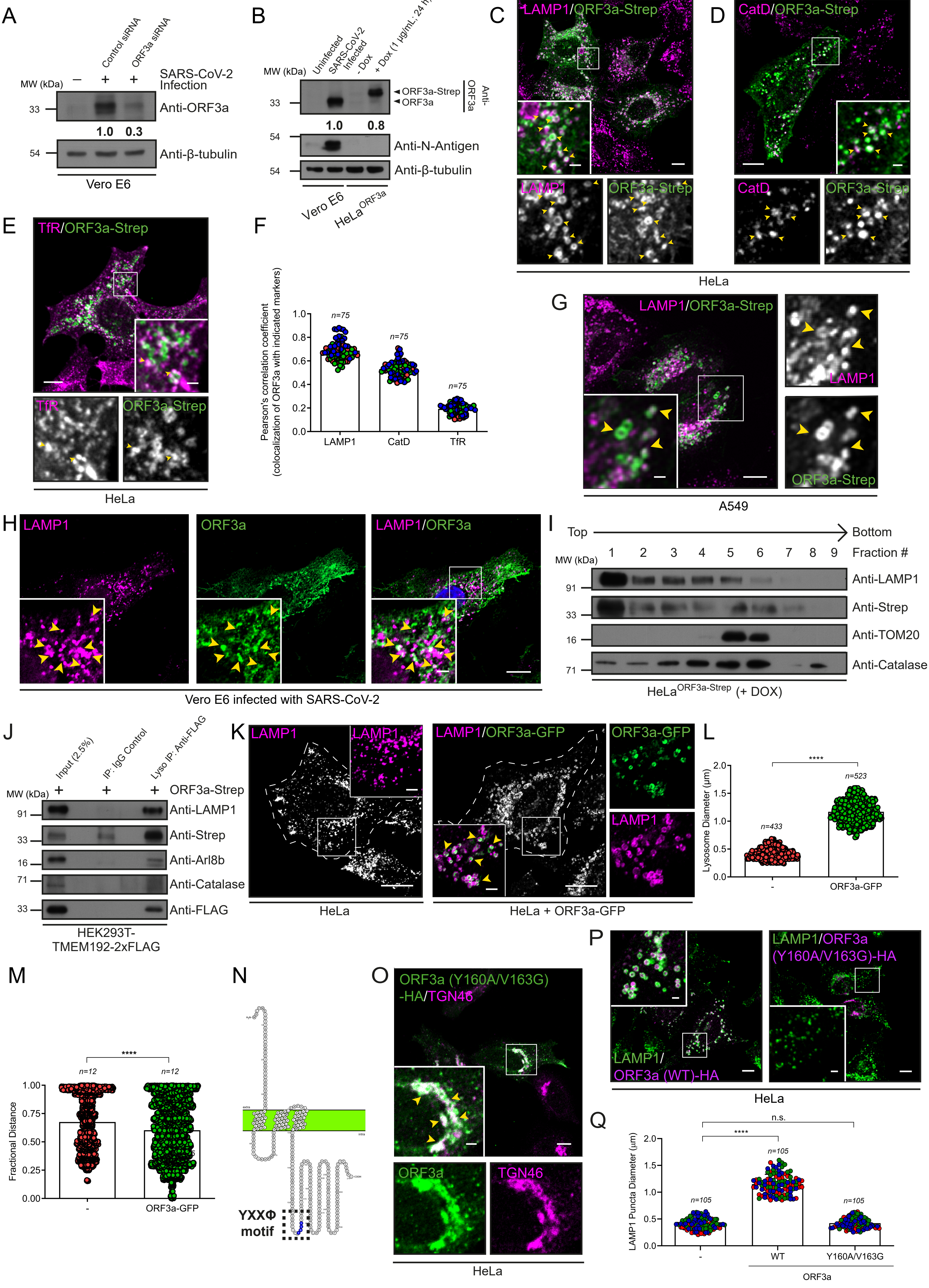

Supplementary Figure S1: ORF3a localizes to lysosomes, and its expression leads to the formation of enlarged perinuclear lysosomes. (A) Lysates of Vero E6 cells infected with SARS-CoV-2 and transfected with control or ORF3a siRNA were IB with indicated antibodies. The values represent densitometric analysis of SARS-CoV-2 ORF3a levels normalized to β-tubulin. **(B)** Lysates of Vero E6 cells uninfected or infected with SARS-CoV-2 and of untreated and Dox-treated $HeLa^{ORF3a\text{-}Strep}$ cells were IB with indicated antibodies. The values represent the densitometric analysis of ORF3a levels normalized to β-tubulin. **(C-E)** Representative confocal images showing HeLa cells expressing ORF3a-Strep stained for indicated organelle markers. **(F)** The PCC quantification of ORF3a-Strep with the indicated markers, n=75 cells examined over three independent experiments. **(G)** Representative confocal images showing A549 cells expressing ORF3a-Strep and stained for LAMP1. **(H)** Representative confocal images of Vero E6 cells infected with SARS-CoV-2 and immunostained for ORF3a and LAMP1. **(I)** Lysosome enrichment was performed on Dox-treated $HeLa^{ORF3a\text{-}Strep}$ cells. Different fractions were IB with indicated antibodies. **(J)** Lysates prepared from HEK293T cells expressing TMEM192-2x-FLAG, transfected with ORF3a-Strep were subjected to LYSO-IP IB using the indicated antibodies. **(K)** Representative super-resolution images show HeLa (wild-type) and ORF3a-GFP-expressing cells immunostained for LAMP1. **(L** and **M)** Quantification of the size of LAMP1-positive compartments (**L**) and distribution of lysosomes within the cell (**M**), n=433 (untransfected) and n=523 (ORF3a-GFP) lysosomes **(L)** and n=12 **(M)** cells examined over three independent experiments, $p<0.0001$. **(N)** Protter-illustration of SARS-CoV-2 ORF3a showing an extracellular N-terminus, three transmembrane domains, and a cytosolic tail. The YXXΦ sorting motif (amino acids 160–163) of ORF3a is highlighted in blue. **(O)** Representative confocal images showing HeLa cells expressing the ORF3a (Y160A/V163G)-HA mutant and immunostained for TGN46. **(P)** Representative confocal images showing HeLa cells expressing the ORF3a (WT)- or (Y160A/V163G)-HA mutant and immunostained for LAMP1. **(Q)** Quantification of the size of LAMP1-positive endosomes in HeLa cells expressing the ORF3a (WT)- or (Y160A/V163G)-HA mutant, n=105 cells examined over three independent experiments, $p<0.0001$ (WT), 0.0812 (Y160A/V163G). Quantified results are presented as mean ± S.D. using unpaired two-tailed Student's *t* test. Scale bars: 10 μm (main); 2 μm (inset).

SUPPLEMENTARY FIGURE S2

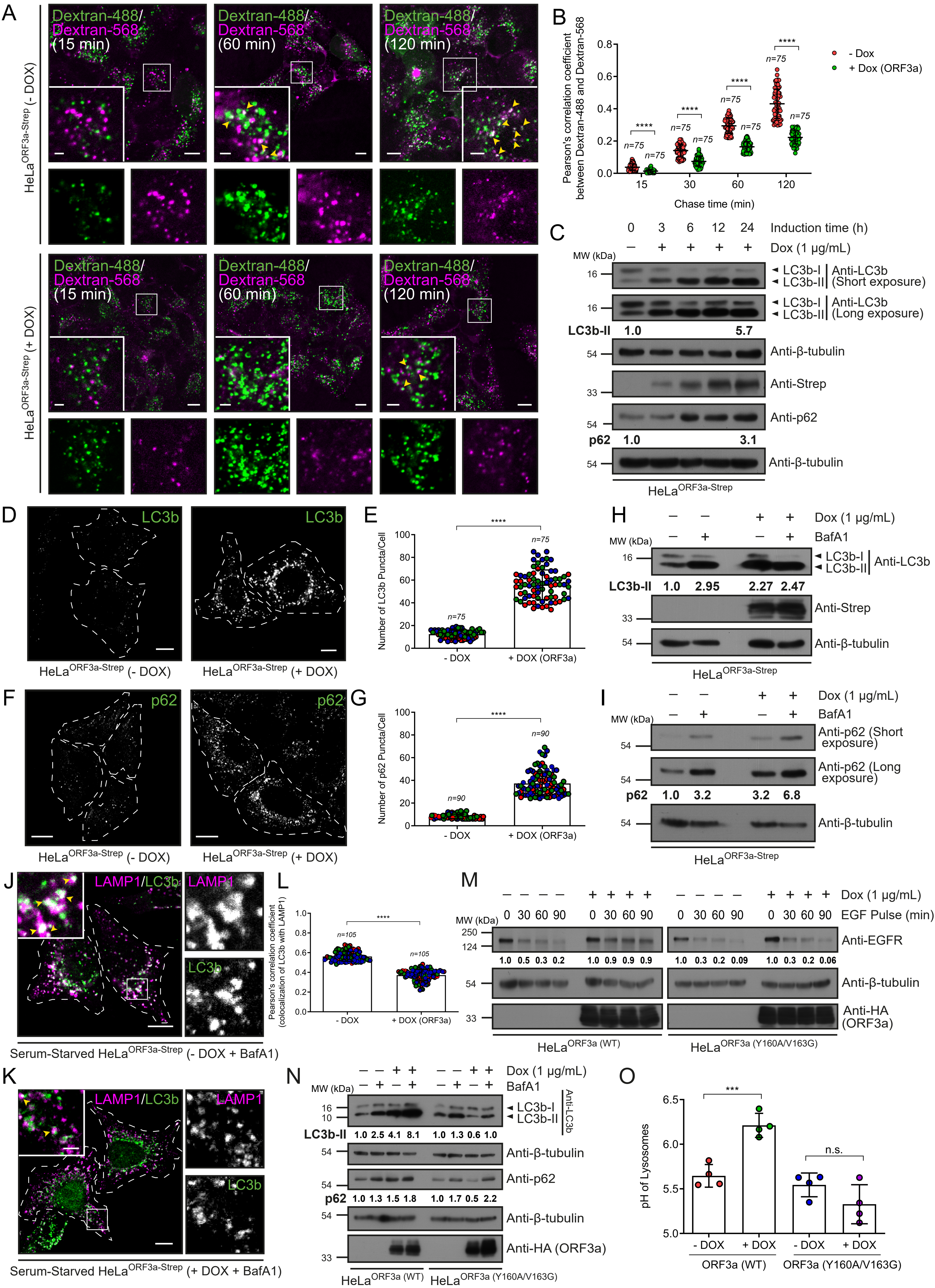

Supplementary Figure S2: ORF3a inhibits autophagic cargo degradation and disrupts the lysosomal pH. (A) Confocal micrographs of dextran uptake experiments performed on untreated and Dox-treated HeLa$^{ORF3a\text{-}Strep}$ cells for the indicated time periods. **(B)** The colocalization of dextran-488-labeled lysosomes with dextran-568-containing vesicles was measured at the indicated time points using PCC, n=75 cells at each time point examined over three independent experiments, *p*<0.0001. **(C)** HeLa$^{ORF3a\text{-}Strep}$ cells were treated with Dox for the indicated time and lysates were IB with indicated antibodies. The values represent the densitometric analysis for LC3b-II and p62 levels normalized to β-tubulin. **(D-G)** Representative confocal micrographs of untreated and Dox-treated HeLa$^{ORF3a\text{-}Strep}$ cells immunostained for LC3b (**D**) and p62 (**F**), respectively. The graphs represent quantification of the puncta number of LC3b (**E**), n=75 and p62 (**G**), n=90 cells examined over three independent experiments, *p*<0.0001. **(H and I)** Untreated and Dox-treated HeLa$^{ORF3a\text{-}Strep}$ cells were treated with bafilomycin-A (BafA1) and lysates were IB for the indicated proteins. The values represent the densitometric analysis of LC3b-II (**H**) and p62 (**I**) levels normalized to β-tubulin. **(J-L)** Untreated (**J**) and Dox-treated (**K**) HeLa$^{ORF3a\text{-}Strep}$ cells were incubated in serum-free medium for 1 h in the presence of BafA1, fixed and immunostained for LC3b and LAMP1. From the confocal images, the colocalization of LC3b with LAMP1 was measured using PCC **(L)**, n=105 cells examined over three independent experiments, *p*<0.0001. **(M)** Untreated and Dox-treated HeLa$^{ORF3a\ (WT)\text{-}HA}$ and HeLa$^{ORF3a\ (Y160A/V163G)\text{-}HA}$ cells were serum-starved and pulsed with EGF for the indicated time periods. Cell lysates were IB with indicated antibodies. The numbers represent densitometric analysis of the EGFR band intensity normalized to the β-tubulin. **(N)** Lysates of HeLa$^{ORF3a\ (WT)\text{-}HA}$ and HeLa$^{ORF3a\ (Y160A/V163G)\text{-}HA}$ cells treated with Dox were IB for the indicated proteins. The values represent the densitometric analysis of LC3b-II and p62 levels normalized to β-tubulin. **(O)** Graph showing the average pH of lysosomes in untreated and Dox-treated HeLa$^{ORF3a\ (WT)\text{-}HA}$ and HeLa$^{ORF3a\ (Y160A/V163G)\text{-}HA}$ cells, *n=3*, *p*=0.0009, 0.1413, respectively. Quantified results are presented as mean ± S.D. using unpaired two-tailed Student's *t* test. Scale bars: 10 μm (main); 2 μm (inset).

SUPPLEMENTARY FIGURE S3

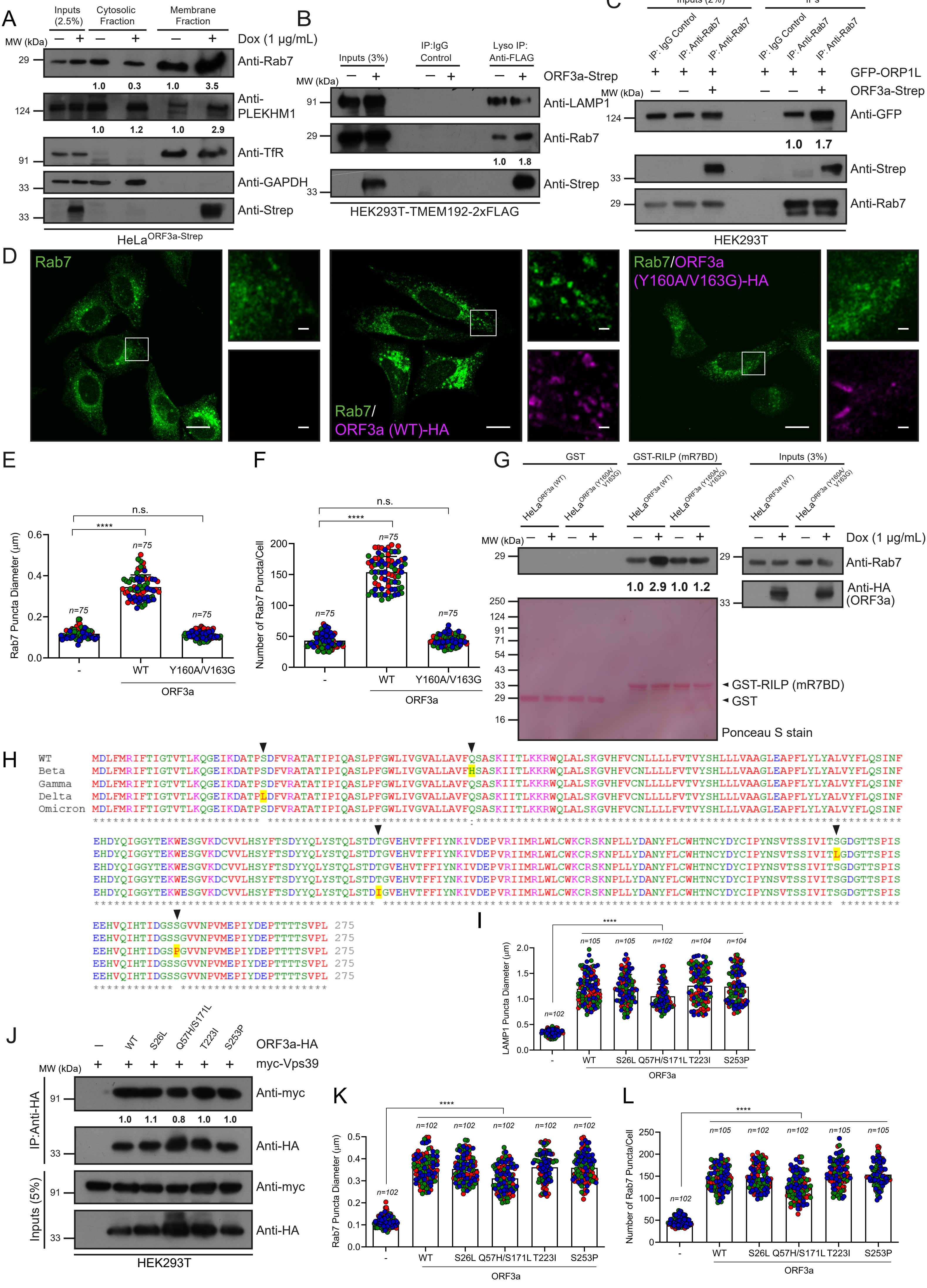

Supplementary Figure S3: ORF3a and its natural variants localize to lysosomes and promote Rab7 activation. (A) Membrane and the cytosol fractions of untreated and Dox-treated HeLa$^{ORF3a\text{-}Strep}$ cells were separated using ultracentrifugation, and were IB with the indicated antibodies. The values represent the densitometric analysis of Rab7 and PLEKHM1 levels. **(B)** Lysates from untransfected and ORF3a-Strep-transfected HEK293T cells expressing TMEM192-2x-FLAG were subjected to LYSO-IP and IB using the indicated antibodies. The values represent the densitometric analysis of the Rab7. **(C)** Lysates of HEK293T cells expressing the indicated proteins were IP with anti-Rab7 antibody-conjugated beads and IB with indicated antibodies. The values represent densitometric analysis of co-immunoprecipitated GFP-ORP1L. **(D)** Representative confocal micrographs of HeLa cells (untransfected) or expressing ORF3a (WT)- or (Y160A/V163G)-HA and immunostained for Rab7. Scale bars: 10 μm (main); 2 μm (inset). **(E** and **F)** Quantification of the size (**E**) and number (**F**) of Rab7-positive endosomes in HeLa cells expressing ORF3a (WT)- or (Y160A/V163G)-HA, n=75 cells examined over three independent experiments. For **(E)**, $p<0.0001$ (WT), 0.0958 (Y160A/V163G); for **(F)**, $p<0.0001$ (WT), 0.756 (Y160A/V163G). **(G)** GST and GST-RILP proteins immobilized on beads were incubated with lysates from untreated and Dox-treated HeLa$^{ORF3a\ (WT)\text{-}HA}$ or HeLa$^{ORF3a\ (Y160A/V163G)\text{-}HA}$ cells. The precipitates were IB with indicated antibodies. The values written represent densitometric analysis of the levels of active Rab7 pulldown. **(H)** Multiple protein sequence alignment of ORF3a (WT) with its naturally occurring variants. The mutations are marked with black arrowheads. **(I)** Quantification of the size of LAMP1-positive compartments in HeLa cells expressing ORF3a (WT) or its natural variants, n=102, 104 or 105 cells examined over three independent experiments, as indicated, $p<0.0001$. **(J)** Lysates of HEK293T cells expressing the indicated proteins were IP with anti-HA antibody-conjugated beads and IB with the indicated antibodies. The values represent the densitometric analysis of co-immunoprecipitated myc-Vps39. **(K** and **L)** Quantification of the size (**K**) and number (**L**) of Rab7-positive endosomes in HeLa cells expressing ORF3a (WT) or its natural variants, n=102 or 105 cells examined over three independent experiments, as indicated, $p<0.0001$. Quantified results are presented as mean ± S.D. using unpaired two-tailed Student's *t* test.

SUPPLEMENTARY FIGURE S4

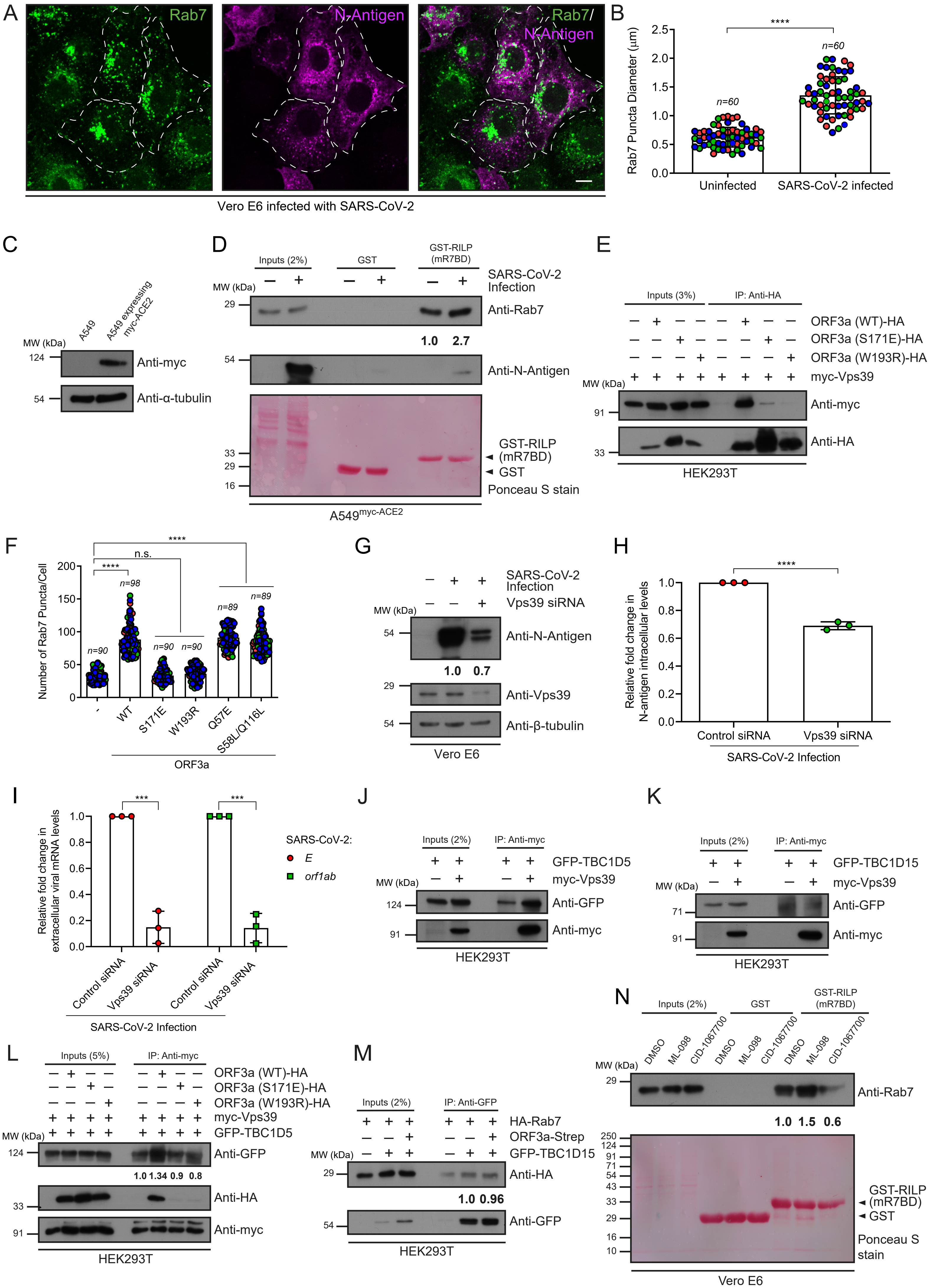

Supplementary Figure S4: The complex of ORF3a, Vps39, and Rab7 GAP TBC1D5 disrupts the interaction between Rab7 and TBC1D5, leading to Rab7 hyperactivation in SARS-CoV-2-infected cells. (A) Representative confocal images showing Vero E6 cells infected with SARS-CoV-2 and immunostained for indicated proteins. Scale bars: 10 µm. **(B)** Quantification of Rab7 puncta size in uninfected and SARS-CoV-2-infected Vero E6 cells, n=60 cells examined over three independent experiments. **(C)** Lysates from wild-type and myc-ACE2 expressing A549 cells were IB with the indicated antibodies. **(D)** GST and GST-RILP proteins immobilized on beads were incubated with lysates from uninfected and SARS-CoV-2-infected A549$^{myc\text{-}ACE2}$ cells. The precipitates were IB with indicated antibodies. The values represent densitometric analysis of the levels of active Rab7 pulldown. **(E)** Lysates of HEK293T cells expressing the indicated proteins were IP using anti-HA-antibody-conjugated beads and IB with indicated antibodies. **(F)** Quantification of Rab7-positive endosomes in HeLa cells expressing indicated ORF3a protein, n=90, 98 or 89 cells examined over three independent experiments, p<0.0001 (WT, Q57E, S58L/Q116L), 0.9286 (S171E), 0.0665 (W193R). **(G)** Lysates of Vero E6 cells infected with SARS-CoV-2 and transfected with control or Vps39 siRNA were IB with indicated antibodies. The values in **(G)** and graph **(H)** represent the densitometric analysis of N-antigen levels normalized to β-tubulin, $n=3$, p<0.0001. **(I)** Relative expression of the *E* and *orf1ab* genes measured in the culture supernatants, $n=3$, p=0.0003 (*E*), 0.0002 (*orf1ab*). **(J** and **K)** Lysates of HEK293T cells expressing the indicated proteins were IP with anti-myc-antibody-conjugated beads and IB with indicated antibodies. **(L)** Lysates of HEK293T cells expressing the indicated proteins were IP with anti-myc-antibody-conjugated beads and IB with indicated antibodies. The values represent densitometric analysis of co-immunoprecipitated GFP-TBC1D5. **(M)** Lysates of HEK293T cells expressing the indicated proteins were IP with anti-GFP-antibody-conjugated beads and IB with the indicated antibodies. The values represent the densitometric analysis of co-immunoprecipitated HA-Rab7. **(N)** GST and GST-RILP proteins immobilized on beads were incubated with lysates of Vero E6 cells treated with respective compound. The precipitates were IB with indicated antibodies. The values represent densitometric analysis of the levels of active Rab7 pulldown.

SUPPLEMENTARY FIGURE S5

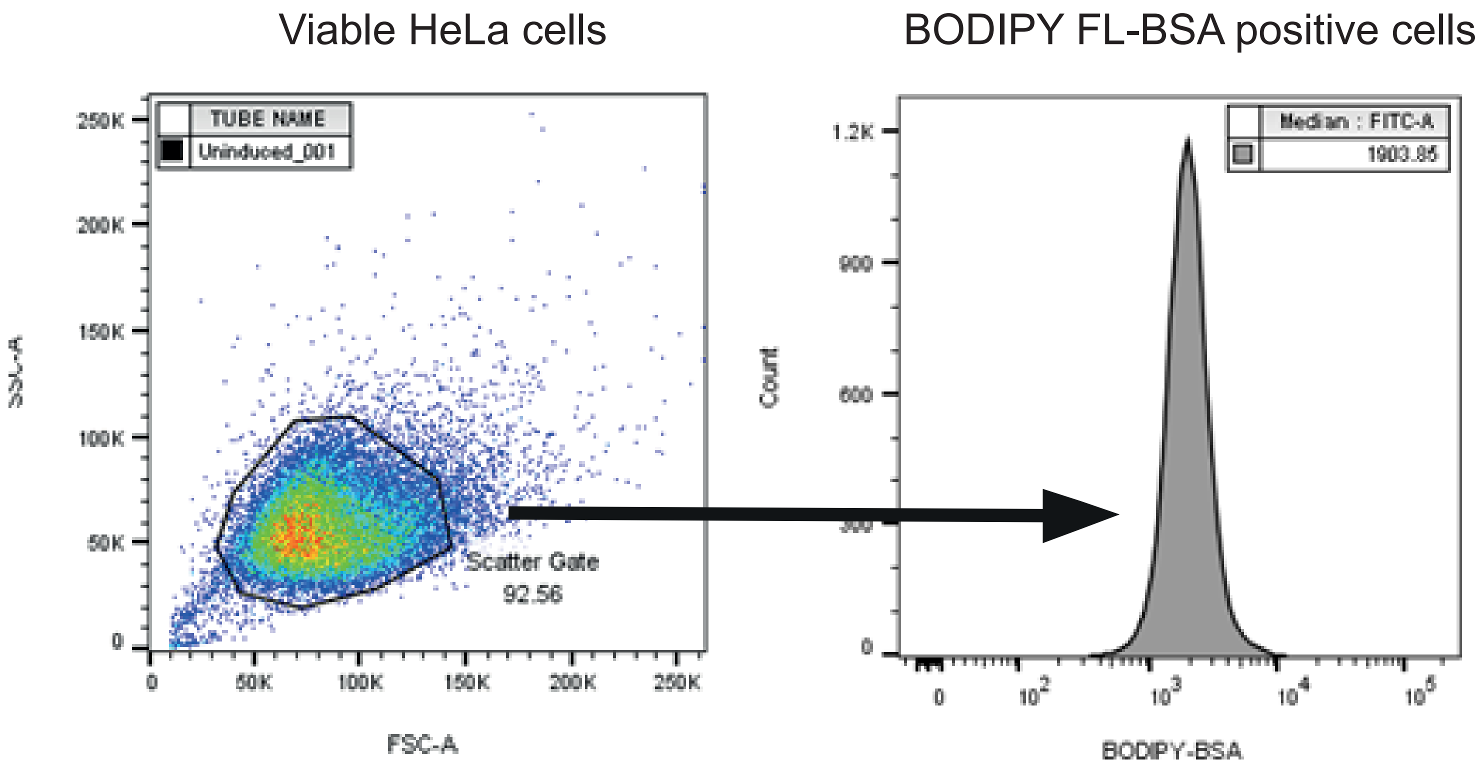

Supplementary Figure S5: Gating strategy for the measurement of BODIPY FL-BSA uptake in HeLa$^{\text{ORF3a-Strep}}$ cells by Flow cytometry. Example of gating strategy used to measure BODIPY FL-BSA fluorescence signal in HeLa$^{\text{ORF3a-Strep}}$ cells. All Flow cytometry analysis were performed on 30,000 cells and viable cells were first gated on a plot of SSC-A versus FSC-A. The data panel presented here corresponds to one of the nine biological replicates of HeLa$^{\text{ORF3a-Strep}}$ cells pulsed with BODIPY FL-BSA for 2 h (Figure 1L).

Supplementary Table I: List of DNA constructs used in this study.

Plasmid Name	Description	Source
Mammalian expression constructs:		
pcDNA3.1(-)	Mammalian expression vector	Invitrogen
pcDNA3.1(-)-ORF3a (WT)-HA	SARS-CoV-2 ORF3a (Wild-type; WT) with C-terminus HA-tag cloned in pcDNA3.1(-)	This study
pcDNA3.1(-)-ORF3a (Q57E)-HA	SARS-CoV-2 ORF3a (Q57E mutant) with C-terminus HA-tag cloned in pcDNA3.1(-)	This study
pcDNA3.1(-)-ORF3a (S58L/Q116L)-HA	SARS-CoV-2 ORF3a (S58L/Q116L mutant) with C-terminus HA-tag cloned in pcDNA3.1(-)	This study
pcDNA3.1(-)-ORF3a (S171E)-HA	SARS-CoV-2 ORF3a (S171E mutant) with C-terminus HA-tag cloned in pcDNA3.1(-)	This study
pcDNA3.1(-)-ORF3a (W193R)-HA	SARS-CoV-2 ORF3a (W193R mutant) with C-terminus HA-tag cloned in pcDNA3.1(-)	This study
pcDNA3.1(-)-ORF3a (S26L)-HA	SARS-CoV-2 ORF3a (S26L mutant) with C-terminus HA-tag cloned in pcDNA3.1(-)	This study
pcDNA3.1(-)-ORF3a (Q57H/S171L)-HA	SARS-CoV-2 ORF3a (Q57H/S171L mutant) with C-terminus HA-tag cloned in pcDNA3.1(-)	This study
pcDNA3.1(-)-ORF3a (T223I)-HA	SARS-CoV-2 ORF3a (T223I mutant) with C-terminus HA-tag cloned in pcDNA3.1(-)	This study
pcDNA3.1(-)-ORF3a (S253P)-HA	SARS-CoV-2 ORF3a (S253P mutant) with C-terminus HA-tag cloned in pcDNA3.1(-)	This study
pcDNA3.1(-)-ORF3a (Y160A/V163G)-HA	SARS-CoV-2 ORF3a (Y160A/V163G mutant) with C-terminus HA-tag cloned in pcDNA3.1(-)	This study
pcDNA3.1(-)-FLAG-PLEKHM1	N-terminus-FLAG-tag-human PLEKHM1 cloned in pcDNA3.1(-)	Described previously (Marwaha et al., 2017)
pcDNA3.1(-)-Arl8b-HA	Human Arl8b with C-terminus HA-tag cloned in pcDNA3.1(-)	Described previously (Marwaha et al., 2017)
pcDNA3.1(-)-FLAG-Rab7	N-terminus-FLAG-tag-human Rab7 cloned in pcDNA3.1(-)	This study
pEBB-HA-Rab7	N-terminus-HA-tag-human Rab7 cloned in pEBB	Gift from Prof. Jason Kinchen (University of Virginia, USA)
pcDNA3.1(-)-HA-Vps39	N-terminus-HA-tag-human Vps39 cloned in pcDNA3.1(-)	Described previously (Khatter et al., 2015)
Vps39-myc	Human Vps39 fused to C-terminal myc-tag	Gift from Prof. Zsófia Simon-Vecsei (Eötvös Loránd University, Hungary)
pEGFP-N1	Mammalian expression vector for C-terminal GFP tag	Clontech
pEGFP-C1	Mammalian expression vector for N-terminal GFP tag	Clontech
pEGFP-N1-ORF3a (WT)	SARS-CoV-2 ORF3a (Wild-type; WT) cloned in pEGFP-N1	This study
pEGFP-N1-ORF3a (S26L)	SARS-CoV-2 ORF3a (S26L mutant) cloned in pEGFP-N1	This study
pEGFP-N1-ORF3a (Q57H/S171L)	SARS-CoV-2 ORF3a (Q57H/S171L mutant) cloned in pEGFP-N1	This study

pEGFP-N1-ORF3a (T223I)	SARS-CoV-2 ORF3a (T223I mutant) cloned in pEGFP-N1	This study
pEGFP-N1-ORF3a (S253P)	SARS-CoV-2 ORF3a (S253P mutant) cloned in pEGFP-N1	This study
GFP-ORP1L	GFP-fused to ORP1L	Gift from Prof. Jacques Neefjes (Leiden University Medical Center, The Netherlands)
GFP-RILP	GFP-fused to RILP	Gift from Prof. Jacques Neefjes (Leiden University Medical Center, The Netherlands)
pEGFP-C1-TBC1D5	Human TBC1D5 cloned in pEGFP-C1	Gift from Prof. Matthew Seaman Lab (University of Cambridge, UK)
pEGFP-C1-TBC1D15	Human TBC1D15 cloned in pEGFP-C1	Gift from Prof. Jacques Neefjes (Leiden University Medical Center, The Netherlands)
LAMP1-GFP	LAMP1 fused to GFP	Gift from Prof. Steve Caplan (University of Nebraska Medical Center, USA)
pEGFP-C1-Rab7	Human Rab7 cloned in pEGFP-C1	Gift from Prof. Steve Caplan (University of Nebraska Medical Center, USA)
ptdTomato-N1	Mammalian expression vector for C-terminal tdTomato tag	Clontech
ptdTomato-N1-Arl8b-tomato	Human Arl8b cloned in ptdTomato-N1	Described previously (Marwaha et al., 2017)
pLVX-EF1alpha-SARS-CoV-2-ORF3a-2xStrep-IRES-Puro	Lentiviral expression of SARS-CoV-2 ORF3a protein with C-terminal Strep tag	Addgene # 141383
pLVX-EF1alpha-eGFP-2xStrep-IRES-Puro	Lentiviral expression of GFP protein with C-terminal Strep tag	Addgene # 141395
pLVX-TetOne-Puro-SARS-CoV-2-ORF3a-2xStrep	Inducible lentiviral expression of SARS-CoV-2 ORF3a protein with C-terminal Strep tag	Gift from Prof. Nevan Krogan (University of California San Francisco, USA)
pLVX-TetOne-PURO-SARS-CoV-2-ORF3a (WT)-HA	Inducible lentiviral expression of SARS-CoV-2 ORF3a (Wild-type; WT) with C-terminal HA tag	This study
pLVX-TetOne-PURO-SARS-CoV-2-ORF3a (Y160A/V163G)-HA	Inducible lentiviral expression of SARS-CoV-2 ORF3a (Y160A/V163G mutant) with C-terminal HA tag	This study
pLVX-TetOne-PURO-SARS-CoV-2-ORF3a (S171E)-HA	Inducible lentiviral expression of SARS-CoV-2 ORF3a (S171E mutant) with C-terminal HA tag	This study
pLVX-TetOne-PURO-SARS-CoV-2-ORF3a (W193R)-HA	Inducible lentiviral expression of SARS-CoV-2 ORF3a (W193R mutant) with C-terminal HA tag	This study
pLVX-TetOne-PURO-GFP-Rab7 (WT)-Rescue	Inducible lentiviral expression of siRNA-resistant Rab7 (Wild-type; WT) with N-terminal GFP tag	This study
pLVX-TetOne-PURO-GFP-Rab7 (Q67L)- Rescue	Inducible lentiviral expression of siRNA-resistant Rab7 (Q67L mutant) with N-terminal GFP tag	This study
pLVX-TetOne-PURO-GFP-Rab7 (T22N)- Rescue	Inducible lentiviral expression of siRNA-resistant Rab7 (T22N mutant) with N-terminal GFP tag	This study
pCDH-CMV-EF1-Puro-myc-ACE2	Lentiviral expression of N-terminal myc-tagged Human ACE2	This study

Str-KDEL-IRES-SBP-mCherry-CTSZ	RUSH construct encoding for the hook protein (Streptavidin-KDEL) and cargo protein (Streptavidin-binding protein-mCherry-Cathepsin Z)	Gift from Prof. Junjie Hu (Institute of Biophysics, Chinese Academy of Sciences, China)
pLJC5-TMEM192-2xFLAG	Human TMEM192 with C-terminus 2xFLAG tag cloned in pLJC5	Addgene # 102929
Bacterial expression constructs:		
pGEX-4T-3 vector	Bacterial expression of GST	GE HealthCare
pGEX-4T-3-mR7BD	Bacterial expression of GST-mR7BD (Rab7 binding domain of murine RILP)	Addgene plasmid #79149

Supplementary Table II: List of antibodies used in this study.

WB: Western Blotting; IF: Immunofluorescence; IP: Immunoprecipitation

S. No.	Antibody (Dilution used for WB: Western Blotting, IF: Immunofluorescence)	Source	Catalogue Number	Clone Number	Lot Number
1.	Mouse anti-Rab7 (WB-1:1000; IF-1:25)	Santa Cruz Biotechnology	sc-376362	B-3	A2121
2.	Rabbit anti-Rab7 (WB-1:1000; IF-1:50)	Cell Signaling Technology	9367	D95F2	1 and 3
3.	Rabbit anti-EGFR (WB-1:1000)	Santa Cruz Biotechnology	sc-03	1005	A2014
4.	Mouse anti-EEA1 (IF-1:200)	BD Biosciences	610457	14/EEA1	3067842
5.	Rabbit anti-EEA1 (IF-1:200)	Cell Signaling Technology	3288	C45B10	8
6.	Rabbit anti-EGFR (IF-1:500)	Thermo Fisher Scientific	MA5-13269	111.6	VL3106561
7.	Mouse anti-Strep (WB-1:10000; IF-1:1000)	Sigma-Aldrich	SAB2702215	GT517	41246
8.	Rabbit anti-HA (WB-1:4000; IF-1:500)	Sigma-Aldrich	H6908	Polyclonal	077M4854V
9.	Mouse anti-HA (WB-1:2000; IF-1:500)	BioLegend	901503	16B12	B242905
10.	Rabbit anti-Giantin (IF-1:2000)	Abcam	ab24586	Polyclonal	GR69388-5
11.	Mouse anti-Vps35 (IF-1:100)	Santa Cruz Biotechnology	sc-374372	B-5	B2316
12.	Rabbit anti-FLAG (WB-1:3000; IF-1:500)	Thermo Fisher Scientific	PA1-984B	Polyclonal	WA309414
13.	Mouse anti-FLAG (WB-1:4000; IF-1:500)	Sigma-Aldrich	F1804	M2	SLBQ6349V
14.	Rabbit anti-TGN46 (IF-1:500)	Abcam	ab50595	Polyclonal	GR283832-6
15.	Mouse anti-α-tubulin (WB-1:5000)	Sigma-Aldrich	T9026	DM1A	137585
16.	Rabbit anti-α-tubulin (WB-1:5000)	Abcam	ab15246	Polyclonal	GR3405100-2
17.	Rabbit anti-Vps11 (WB-1:2000)	Abcam	ab170869	EPR10345	YK010208S
18.	Rabbit anti-Vps18 (WB-1:2000)	Abcam	ab178416	EPR13378	GR141390-8
19.	Rabbit anti-Vps33a (WB-1:2000)	ProteinTech	16896-1-AP	Polyclonal	8325
20.	Mouse anti-Vps39 (WB-1:250)	Santa Cruz Biotechnology	sc-514762	C-5	G2417
21.	Rabbit anti-Vps41 (WB-1:1000)	Abcam	ab181078	EPR13268	GR164325-1

22.	Mouse anti-Vps41 (WB-1:500)	Santa Cruz Biotechnology	sc-377271	E-10	B2718
23.	Mouse anti-LAMP1 (WB-1:5000; IF-1:500)	BD Biosciences	555798	H4A3	9046526
24.	Rabbit anti-PLEKHM1 (WB-1:3000; IF-1:500)	Custom-made	Gift from Prof. Paul Odgren (University of Massachusetts Medical School, USA)	-	-
25.	Mouse anti-β-tubulin (WB-1:5000)	Sigma-Aldrich	T4026	TUB 2.1	107M4801V
26.	Rabbit anti-N-antigen (WB-1:10000; IF-1:2000)	Rockland Immunochemicals	200-401-A50	Polyclonal	46527
27.	Mouse anti-myc (WB-1:1000)	Santa Cruz Biotechnology	sc-40	9E10	J0220
28.	Mouse anti-CI-M6PR (IF-1:500)	Abcam	ab2733	2G11	GR3394002-1
29.	Rabbit anti-CI-M6PR (IF-1:500)	Abcam	ab124767	EPR6599	GR3366649
30.	Mouse anti-Rab5 (WB-1:1000)	BD Bioscience	610281	15/Rab5	1067628
31.	Rabbit anti-LAMP1 (WB-1:5000; IF-1:1000)	Abcam	ab24170	Polyclonal	GR294265-4
32.	Rabbit anti-Arl8b (WB-1:1000; IF-1:30)	Cell Signaling Technology	56085	Polyclonal	1
33.	Mouse anti-TOM20 (WB-1:1000)	Santa Cruz Biotechnology	sc-17764	F-10	B2018
34.	Rabbit anti-LC3b (WB-1:1000)	Sigma-Aldrich	L7543	Polyclonal	084M4798V
35.	Rabbit anti-TBC1D5 (WB-1:2000)	Abcam	ab203896	Polyclonal	1002271-2
36.	Rabbit anti-Cathepsin D (WB-1:1000; IF-1:250)	Abcam	ab75852	EPR3057Y	1014146-16
37.	Rabbit anti-Catalase (WB-1:1000)	Cell Signaling Technology	12980	D4P7B	1
38.	Rabbit anti-TfR (WB-1:1000; IF-1:250)	Abcam	ab84036	Polyclonal	GR3400985-1
39.	Rabbit anti-Arl8b (WB-1:1000; IF-1:30)	Custom-made	Described previously (Garg et al., 2011)	-	-
40.	Rabbit anti-LC3 (IF-1:1000)	MBL International Corporation	PM036	Polyclonal	35
41.	Rabbit anti-p62 (IF-1:1000)	MBL International Corporation	PM045	Polyclonal	21
42.	Rabbit anti-LC3B (WB-1:2000)	Cell Signaling Technology	3868	D11	11
43.	Rabbit anti-p62 (WB-1:1000)	Cell Signaling Technology	5114	Polyclonal	4

44.	Mouse anti-GAPDH (WB-1:1000)	Santa Cruz Biotechnology	sc-166574	H-12	J0721
45.	Mouse anti-GFP (WB-1:1000)	Santa Cruz Biotechnology	sc-9996	B-2	C0117
46.	Rabbit anti-ORF3a (WB-1:2000; IF-1:100)	Cell Signaling Technology	34340	Polyclonal	1
47.	Rabbit anti-Spike (WB-1:500)	ABclonal	A20022	ARC2373	10413
48.	Rabbit IgG-conjugated agarose beads	Sigma-Aldrich	A2909	-	-
49.	Mouse IgG-conjugated agarose beads	Sigma-Aldrich	A0919	-	-
50.	Anti-FLAG affinity gel (IP-13 µL slurry)	BioLegend	651503	L5	B233846
51.	Anti-HA affinity gel (IP-13 µL slurry)	Sigma-Aldrich	A2095	HA-7	026M4810V
52.	Anti-myc affinity gel (IP-13 µL slurry)	Sigma-Aldrich	A7470	Polyclonal	093M4823
53.	Anti-GFP affinity gel (IP-13 µL slurry)	Santa Cruz Biotechnology	sc-9996-AC	B-2	H1321 and C0722
54.	Mouse anti-Rab7 conjugated agarose beads (IP-13 µL slurry)	Santa Cruz Biotechnology	sc-376362-AC	B-3	I0916
55.	Alexa-Fluor 488-conjugated goat anti-rabbit IgG (IF-1:500)	Thermo Fisher Scientific	A11034	Polyclonal	1705912
56.	Alexa-Fluor 568-conjugated goat anti-rabbit IgG (IF-1:500)	Thermo Fisher Scientific	A11036	Polyclonal	1924788
57.	Alexa-Fluor 488-conjugated goat anti-mouse IgG (IF-1:500)	Thermo Fisher Scientific	A11029	Polyclonal	1745855
58.	Alexa-Fluor 568-conjugated goat anti-mouse IgG (IF-1:500)	Thermo Fisher Scientific	A11031	Polyclonal	2124366
59.	Alexa-Fluor 633-conjugated goat anti-rabbit IgG (IF-1:500)	Thermo Fisher Scientific	A21245	Polyclonal	56675A
60.	Alexa-Fluor 633-conjugated goat anti-mouse IgG (IF-1:500)	Thermo Fisher Scientific	A21236	Polyclonal	400836
61.	HRP-conjugated goat anti-rabbit IgG (WB-1:5000)	Jackson ImmunoResearch	111-035-144	Polyclonal	133672
62.	HRP-conjugated goat anti-mouse IgG (WB-1:5000)	Jackson ImmunoResearch	115-035-166	Polyclonal	155426 and 152078
