# Supplementary material for: SARS-CoV-2 virulence factor ORF3a blocks lysosome function by modulating TBC1D5-dependent Rab7 GTPase cycle
Source: Nat Commun. 2024 Mar 6;15:2053. doi: 10.1038/s41467-024-46417-2 (PMC10918171; doi:10.1038/s41467-024-46417-2)
Supplement: Supplementary file 3 — Description of Additional Supplementary Files [file 41467_2024_46417_MOESM3_ESM.pdf]

Description of Additional Supplementary Files

File Name: Supplementary Movie 1
Description: Time-lapse imaging of untreated (-Dox) and doxycycline-treated (+Dox) $HeLa^{ORF3a\text{-}Strep}$ cells expressing GFPRab7 for The FRAP assay. The inset shows GFP-Rab7 endosomes subjected to photobleaching, which were analyzed for recovery of the GFP-Rab7 fluorescence signal. The videos were captured at 2.04 frames per second with no time interval between the frames. The video is shown at 7 frames per second (the total number of frames displayed is 200). The time-lapse images obtained from these videos are shown in Fig. 3A. Scale bar: 10 µm.

File Name: Supplementary Movie 2
Description: Time-lapse imaging of untreated (-Dox) and doxycycline-treated (+Dox) $HeLa^{ORF3a\text{-}Strep}$ cells expressing GFPRab7 and Arl8b-tomato. The inset shows the GFP-Rab7-positive endosomes and Arl8btomato-positive endosomes scored for fusion events. The videos were captured at 2.04 frames per second with no time interval between the frames. The video is shown at 5 frames per second (the total number of frames displayed is 200). The time-lapse images of the insets of these videos are shown in Fig. 7E. Scale bar: 10 µm.
