# Supplementary material for: SARS-CoV-2 virulence factor ORF3a blocks lysosome function by modulating TBC1D5-dependent Rab7 GTPase cycle
Source: Nat Commun. 2024 Mar 6;15:2053. doi: 10.1038/s41467-024-46417-2 (PMC10918171; doi:10.1038/s41467-024-46417-2)
Supplement: Supplementary file 6 — Reporting Summary [file 41467_2024_46417_MOESM6_ESM.pdf]

nature portfolio

Corresponding author(s): Amit Tuli

Last updated by author(s): Feb 20, 2024

Reporting Summary

Nature Portfolio wishes to improve the reproducibility of the work that we publish. This form provides structure for consistency and transparency in reporting. For further information on Nature Portfolio policies, see our Editorial Policies and the Editorial Policy Checklist.

Statistics

For all statistical analyses, confirm that the following items are present in the figure legend, table legend, main text, or Methods section.

n/a	Confirmed	
☐	☒	The exact sample size (*n*) for each experimental group/condition, given as a discrete number and unit of measurement
☐	☒	A statement on whether measurements were taken from distinct samples or whether the same sample was measured repeatedly
☐	☒	The statistical test(s) used AND whether they are one- or two-sided<br>*Only common tests should be described solely by name; describe more complex techniques in the Methods section.*
☒	☐	A description of all covariates tested
☒	☐	A description of any assumptions or corrections, such as tests of normality and adjustment for multiple comparisons
☐	☒	A full description of the statistical parameters including central tendency (e.g. means) or other basic estimates (e.g. regression coefficient) AND variation (e.g. standard deviation) or associated estimates of uncertainty (e.g. confidence intervals)
☐	☒	For null hypothesis testing, the test statistic (e.g. *F*, *t*, *r*) with confidence intervals, effect sizes, degrees of freedom and *P* value noted<br>*Give P values as exact values whenever suitable.*
☒	☐	For Bayesian analysis, information on the choice of priors and Markov chain Monte Carlo settings
☒	☐	For hierarchical and complex designs, identification of the appropriate level for tests and full reporting of outcomes
☐	☒	Estimates of effect sizes (e.g. Cohen's *d*, Pearson's *r*), indicating how they were calculated

Our web collection on statistics for biologists contains articles on many of the points above.

Software and code

Policy information about availability of computer code

Data collection	Zen Black 2012 (Zeiss) was used for Carl Zeiss 710 confocal laser scanning microscope.<br>ZEN 2012 v. 8.0.1.273 (ZEISS) was used for LSM 980 Elyra 7 super-resolution microscope.<br>Tecan infinite control 2.0.10.0 for pH measurement.<br>BD FACS Diva software v.8.0.1 (BD Biosciences) for Flow cytometry.<br>CFX96 Real-time system (Bio-Rad) for quantitative real-time PCR.
Data analysis	Data analysis was done using Fiji v. 2.1.0 (NIH). For analysis of particle count and size from confocal micrographs, "analyze particle tool" function of Fiji software was used. For measurement of multivesicular and multilamellar compartment's diameter from TEM images, "line tool" function of Fiji software was used. For measurement of area, "free hand tool and "measure tool" functions of Fiji software was used. For measurement of fractional distance, "plot profile tool", "free-hand selection tool" and "clear outside tool" function of Fiji software was used. Trackmate v. 6.0.1 plugin of Fiji software was used for single-particle tracking. JACoP plugin of Fiji software was used to measure the Pearson’s colocalization coefficient (PCC).<br>BD FlowJo v. 10.0.1. was used to analyze flow-cytometry related experiments.<br>Statistics and graphing was done using Prism v. 8 (GraphPad) and Excel 2013 (Microsoft).<br>All figures were assembled using Fiji v. 2.1.0 (NIH), Adobe Illustrator v. 24.3 and Adobe Photoshop v.22.0.0.<br>BioRender.com (Agreement Number: QT26GKEQTR) was used to create Figure 9.<br>Protter (http://wlab.ethz.ch/protter/#) was used to create illustration shown in Supplementary Figure S1 (N).<br>Clustal Omega (https://www.ebi.ac.uk/jdispatcher/msa/clustalo) was used for multiple sequence alignment shown in Figure S2 (H).

For manuscripts utilizing custom algorithms or software that are central to the research but not yet described in published literature, software must be made available to editors and reviewers. We strongly encourage code deposition in a community repository (e.g. GitHub). See the Nature Portfolio guidelines for submitting code & software for further information.

Data

Policy information about availability of data

All manuscripts must include a data availability statement. This statement should provide the following information, where applicable:

- Accession codes, unique identifiers, or web links for publicly available datasets
- A description of any restrictions on data availability
- For clinical datasets or third party data, please ensure that the statement adheres to our policy

All relevant information supporting the findings of this study is presented in the manuscript and supplementary materials. A source file comprising raw data and western blot images that have not been cropped is included in the manuscript.

Research involving human participants, their data, or biological material

Policy information about studies with human participants or human data. See also policy information about sex, gender (identity/presentation), and sexual orientation and race, ethnicity and racism.

Reporting on sex and gender	N/A
Reporting on race, ethnicity, or other socially relevant groupings	N/A
Population characteristics	N/A
Recruitment	N/A
Ethics oversight	N/A

Note that full information on the approval of the study protocol must also be provided in the manuscript.

Field-specific reporting

Please select the one below that is the best fit for your research. If you are not sure, read the appropriate sections before making your selection.

☒ Life sciences ☐ Behavioural & social sciences ☐ Ecological, evolutionary & environmental sciences

For a reference copy of the document with all sections, see nature.com/documents/nr-reporting-summary-flat.pdf

Life sciences study design

All studies must disclose on these points even when the disclosure is negative.

Sample size	The figure legends of the relevant figures list all sample sizes in detail. Sample sizes were determined based on the estimates from preliminary experiments and similar studies (Kumar G. et al., Nature Communications, 1540 (2022), Willett R. et al., Nature Communications, 1580 (2017), and Marwaha R. et al., Journal of Cell Biology, 216 (2017)) so that reasonable statistical analysis could be conducted. A minimum of three biological replicates were taken for each experimental set, unless otherwise mentioned.
Data exclusions	Any transfected cell expressing an excessively high amount of proteins that visibly altered the cell morphology or exhibited saturated signal intensities was excluded from the analysis. Also, cells with two nuclei and non-uniform morphology were excluded from analysis.
Replication	All the data presented were from biological replicates. Each experiment was repeated multiple times independently, as indicated in the figure legends, and was reproducible. Some of the experiments were replicated in multiple cell lines. All attempts at replication were successful.
Randomization	The randomization of different experimental groups was irrelevant because all of the experiments were conducted on a uniform biological sample, i.e., eukaryotic cell lines obtained from a commercial source (ATCC). Furthermore, all the cells were treated or imaged, and they were finally analyzed in the same way.
Blinding	Blinding was not possible because the same investigators were responsible for the experiment preparation and further analysis of the data. Further, the experimenters had no preconceptions while analyzing the results.

Reporting for specific materials, systems and methods

We require information from authors about some types of materials, experimental systems and methods used in many studies. Here, indicate whether each material, system or method listed is relevant to your study. If you are not sure if a list item applies to your research, read the appropriate section before selecting a response.

Materials & experimental systems

n/a	Involved in the study
☐	☒ Antibodies
☐	☒ Eukaryotic cell lines
☒	☐ Palaeontology and archaeology
☒	☐ Animals and other organisms
☒	☐ Clinical data
☒	☐ Dual use research of concern
☒	☐ Plants

Methods

n/a	Involved in the study
☒	☐ ChIP-seq
☐	☒ Flow cytometry
☒	☐ MRI-based neuroimaging

Antibodies

Antibodies used

All of the antibodies used in this study are listed in Supplementary Table S2, which includes all relevant information about each antibody.

Validation

The validation statements can be found on the supplier's website using the links provided below. The antibodies successfully detected proteins with their expected molecular weights. The specificity of the antibodies against Rab7, Vps39, and ORF3a were validated in this manuscript.

1. Mouse anti-Rab7 antibody, SCBT was validated by the supplier. https://www.scbt.com/p/rab-7-antibody-b-3. We also validated the antibody in this study and has been previously shown in other publications (PMID: 35314681, 36506095, 28325809, 36282215).
2. Rabbit anti-Rab7 antibody, CST was validated by the supplier. https://www.cellsignal.com/products/primary-antibodies/rab7-d95f2-xp-rabbit-mab/9367. It has been previously shown in other publications (PMID: 35314681, 25908847).
3. Rabbit anti-EGFR antibody, SCBT was validated by the supplier. https://www.citeab.com/antibodies/790856-sc-03-egfr-1005. It has been previously shown in other publications (PMID: 37949862, 36753044, 37598181).
4. Mouse anti-EEA1 antibody, BD Biosciences was validated by the supplier. https://www.bdbiosciences.com/en-us/products/reagents/microscopy-imaging-reagents/immunofluorescence-reagents/purified-mouse-anti-eea1.610457. It has been previously shown in other publications (PMID: 28325809, 25908847, 36282215).
5. Rabbit anti-EEA1 antibody, CST was validated by the supplier. https://www.cellsignal.com/products/primary-antibodies/eea1-c45b10-rabbit-mab/3288. It has been previously shown in other publications (PMID: 36282215, 38126335, 38123554).
6. Rabbit anti-EGFR antibody, Thermo Fisher Scientific was validated by the supplier. https://www.thermofisher.com/antibody/product/EGFR-Antibody-clone-111-6-Monoclonal/MA5-13269. It has been previously shown in other publications (PMID: 32200349, 31338842, 31604911).
7. Mouse anti-Strep antibody, Sigma-Aldrich was validated by the supplier. https://www.sigmaaldrich.com/IN/en/product/sigma/sab2702215. It has been previously shown in other publications (PMID: 33545052, 31932609).
8. Rabbit anti-HA antibody, Sigma-Aldrich was validated by the supplier. https://www.sigmaaldrich.com/IN/en/product/sigma/h6908. It has been previously shown in other publications (PMID: 35314681, 36282215).
9. Mouse anti-HA antibody, BioLegend was validated by the supplier. https://www.biolegend.com/fr-fr/products/purified-anti-ha-11-epitope-tag-antibody-11374?GroupID=GROUP26. It has been previously shown in other publications (PMID: 35314681, 34103528, 33086052).
10. Rabbit anti-Giantin antibody, Abcam was validated by the supplier. https://www.citeab.com/antibodies/733073-ab24586-anti-giantin-antibody. It has been previously shown in other publications (PMID: 36282215, 36804936, 34798070).
11. Mouse anti-Vps35 antibody, Santa Cruz Biotechnology was validated by the supplier. https://www.scbt.com/p/vps35-antibody-b-5. It has been previously shown in other publications (PMID: 36282215, 36810735, 35993307).
12. Rabbit anti-FLAG antibody, Thermo Fisher Scientific was validated by the supplier. https://www.thermofisher.com/antibody/product/DYKDDDDK-Tag-Antibody-Polyclonal/PA1-984B. It has been previously shown in other publications (PMID: 36763502, 33966597, 36244455).
13. Mouse anti-FLAG antibody, Sigma-Aldrich was validated by the supplier. https://www.sigmaaldrich.com/IN/en/product/sigma/f1804. It has been previously shown in other publications (PMID: 35314681, 28325809).
14. Rabbit anti-TGN46 antibody, Abcam was validated by the supplier. https://www.abcam.com/products/primary-antibodies/tgn46-antibody-ab50595.html. It has been previously shown in other publications (PMID: 35235793, 35024770).
15. Mouse anti-α-tubulin antibody, Sigma-Aldrich was validated by the supplier. https://www.sigmaaldrich.com/IN/en/product/sigma/t9026. It has been previously shown in other publications (PMID: 28325809, 20534674).
16. Rabbit anti-α-tubulin antibody, Abcam was validated by the supplier. https://www.abcam.com/products/primary-antibodies/alpha-tubulin-antibody-microtubule-marker-ab15246.html. It has been previously shown in other publications (PMID: 35106468, 35203399).
17. Rabbit anti-Vps11 antibody, Abcam was validated by the supplier. https://www.abcam.com/products/primary-antibodies/vps11-antibody-epr10345-ab170869.html. It has been previously shown in other publications (PMID: 28325809, 25908847).
18. Rabbit anti-Vps18 antibody, Abcam was validated by the supplier. https://www.abcam.com/products/primary-antibodies/vps18-antibody-epr13378-n-terminal-ab178416.html. It has been previously shown in other publications (PMID: 28325809, 29084291).
19. Rabbit anti-Vps33a antibody, ProteinTech was validated by the supplier. https://www.ptglab.com/products/VPS33A-Antibody-16896-1-AP.html. It has been previously shown in other publications (PMID: 28325809, 25908847).
20. Mouse anti-Vps39 antibody, Santa Cruz Biotechnology was validated by the supplier. https://www.scbt.com/p/vps39-antibody-c-5. We also validated the antibody in this study and has been previously shown in other publications (PMID: 35771772, 36640308).
21. Rabbit anti-Vps41 antibody, Abcam was validated by the supplier. https://www.abcam.com/products/primary-antibodies/vps41-antibody-epr13268-ab181078.html. It has been previously shown in other publications (PMID: 28325809, 29084291).
22. Mouse anti-Vps41 antibody, Santa Cruz Biotechnology was validated by the supplier. https://www.scbt.com/p/vps41-antibody-

e-10. It has been previously shown in other publications (PMID: 28325809, 25908847).
23. Mouse anti-LAMP1 antibody, BD Biosciences was validated by the supplier. https://www.fishersci.com/shop/products/anti-cd107a-clone-h4a3-bd/BDB555798. It has been previously shown in other publications (PMID: 35314681, 28325809, 25908847, 36282215).
24. Rabbit anti-PLEKHM1 antibody, Custom-made and gift from Prof. Paul Odgren (University of Massachusetts Medical School, USA). PMID: 25992615. It has been previously shown in other publications (PMID: 28325809, 36282215).
25. Mouse anti-β-tubulin antibody, Sigma-Aldrich was validated by the supplier. https://www.sigmaaldrich.com/IN/en/product/sigma/t4026. It has been previously shown in other publications (PMID: 35314681, 21898690).
26. Rabbit anti-N-antigen antibody, Rockland Immunochemicals was validated by the supplier. https://www.rockland.com/categories/primary-antibodies/sars-nucleocapsid-protein-antibody-200-401-A50/. It has been previously shown in other publications (PMID: 35250984, 35020534).
27. Mouse anti-myc antibody, Santa Cruz Biotechnology was validated by the supplier. https://www.scbt.com/p/c-myc-antibody-9e10. It has been previously shown in other publications (PMID: 37620393, 37628607).
28. Mouse anti-CI-M6PR antibody, Abcam was validated by the supplier. https://www.abcam.com/products/primary-antibodies/m6pr-cation-independent-antibody-2g11-ab2733.html. It has been previously shown in other publications (PMID: 36282215, 35449600, 35314489).
29. Rabbit anti-CI-M6PR antibody, Abcam was validated by the supplier. https://www.abcam.com/products/primary-antibodies/m6pr-cation-independent-antibody-epr6599-ab124767.html. It has been previously shown in other publications (PMID: 36282215, 35318322).
30. Mouse anti-Rab5 antibody, BD Biosciences was validated by the supplier. https://www.bdbiosciences.com/en-sg/products/reagents/microscopy-imaging-reagents/immunofluorescence-reagents/Purified-Mouse-Anti-Rab5.610281. It has been previously shown in other publications (PMID: 35314681, 11687655).
31. Rabbit anti-LAMP1 antibody, Abcam was validated by the supplier. https://www.abcam.com/products/primary-antibodies/lamp1-antibody-lysosome-marker-ab24170.html. It has been previously shown in other publications (PMID: 35314681, 28325809, 36282215).
32. Rabbit anti-Arl8b antibody, Cell Signaling Technology was validated by the supplier. https://www.cellsignal.com/products/primary-antibodies/arl8b-antibody/56085. It has been previously shown in other publications (PMID: 35314681, 36282215).
33. Mouse anti-TOM20 antibody, Santa Cruz Biotechnology was validated by the supplier. https://www.scbt.com/p/tom20-antibody-f-10. It has been previously shown in other publications (PMID: 35314681, 37108539).
34. Rabbit anti-LC3b antibody, Sigma-Aldrich was validated by the supplier. https://www.sigmaaldrich.com/IN/en/product/sigma/l7543. It has been previously shown in other publications (PMID: 23691463, 20657169).
35. Rabbit anti-TBC1D5 antibody, Abcam was validated by the supplier. https://www.abcam.com/products/primary-antibodies/tbc1d5-antibody-ab203896.html. It has been previously shown in other publications (PMID: 32521275).
36. Rabbit anti-Cathepsin D antibody, Abcam was validated by the supplier. https://www.abcam.com/products/primary-antibodies/cathepsin-d-antibody-epr3057y-ab75852.html. It has been previously shown in other publications (PMID: 35314681, 36282215).
37. Rabbit anti-Catalase antibody, Cell Signaling Technology was validated by the supplier. https://www.cellsignal.com/products/primary-antibodies/catalase-d4p7b-xp-rabbit-mab/12980. It has been previously shown in other publications (PMID: 35314681, 38034364).
38. Rabbit anti-TfR antibody, Abcam was validated by the supplier. https://www.abcam.com/products/primary-antibodies/transferrin-receptor-antibody-ab84036.html. It has been previously shown in other publications (PMID: 35314681, 36282215).
39. Rabbit anti-Arl8b antibody, Custom-made and previously used in other publications (PMID: 21802320, 35314681, 28325809, 36282215)
40. Rabbit anti-LC3 antibody, MBL International Corporation was validated by the supplier. https://www.mblbio.com/bio/g/dtl/A/?pcd=PM036. It has been previously shown in other publications (PMID: 35314681, 31822666).
41. Rabbit anti-p62 antibody, MBL International Corporation was validated by the supplier. https://www.mblbio.com/bio/g/dtl/A/?pcd=PM045. It has been previously shown in other publications (PMID: 32080200, 31896748).
42. Rabbit anti-LC3b antibody, Cell Signaling Technology was validated by the supplier. https://www.cellsignal.com/products/primary-antibodies/lc3b-d11-xp-rabbit-mab/3868. It has been previously shown in other publications (PMID: 35314681, 28325809).
43. Rabbit anti-p62 antibody, Cell Signaling Technology was validated by the supplier. https://www.cellsignal.com/products/primary-antibodies/sqstm1-p62-antibody/5114. It has been previously shown in other publications (PMID: 37847564, 38097554).
44. Mouse anti-GAPDH antibody, Santa Cruz Biotechnology was validated by the supplier. https://www.scbt.com/p/gapdh-antibody-h-12. It has been previously shown in other publications (PMID: 35314681, 25908847, 36282215).
45. Mouse anti-GFP antibody, Santa Cruz Biotechnology was validated by the supplier. https://www.scbt.com/p/gfp-antibody-b-2. It has been previously shown in other publications (PMID: 28325809, 36282215).
46. Rabbit anti-ORF3a antibody, Cell Signaling Technology was validated by the supplier. https://www.cellsignal.com/products/primary-antibodies/sars-cov-2-orf3a-antibody/34340. We also validated the antibody in this study and has been previously shown in other publication (PMID: 38005906).
47. Rabbit anti-Spike antibody, ABclonal was validated by the supplier. https://abclonal.com/catalog-antibodies/SARSCoV2SpikeS1mAbBSAandglycerolfree/A20022. It has been previously shown in other publications (PMID: 34239064, 34665481).
48. Rabbit IgG-conjugated agarose beads Sigma-Aldrich was validated by the supplier. https://www.sigmaaldrich.com/IN/en/product/sigma/a2909. It has been previously shown in other publications (PMID: 35314681).
49. Mouse IgG-conjugated agarose beads Sigma-Aldrich was validated by the supplier. https://www.sigmaaldrich.com/IN/en/product/sigma/a0919. It has been previously shown in other publications (PMID: 35314681).
50. Anti-FLAG antibody affinity gel, BioLegend was validated by the supplier. https://www.biolegend.com/fr-lu/products/anti-dykddddk-tag-l5-affinity-gel-7554?GroupID=BLG11431. It has been previously shown in other publications (PMID: 35314681).
51. Anti-HA antibody affinity gel, Sigma-Aldrich was validated by the supplier. https://www.sigmaaldrich.com/IN/en/product/sigma/a2095. It has been previously shown in other publications (PMID: 35314681).
52. Anti-myc antibody affinity gel, Sigma-Aldrich was validated by the supplier. https://www.sigmaaldrich.com/IN/en/product/sigma/a7470. It has been previously shown in other publications (PMID: 24135613, 22157752).
53. Anti-GFP antibody affinity gel, Santa Cruz Biotechnology was validated by the supplier. https://www.scbt.com/p/gfp-antibody-b-2. It has been previously shown in other publications (PMID: 28325809, 36282215).
54. Mouse anti-Rab7 antibody conjugated agarose beads, Santa Cruz Biotechnology was validated by the supplier. https://

www.scbt.com/p/rab-7-antibody-b-3. We also validated the antibody in this study and has been previously shown in other publications (PMID: 35314681, 36506095, 28325809).
55. Alexa-Fluor 488-conjugated goat anti-rabbit IgG, Thermo Fisher Scientific was validated by the supplier. https://www.thermofisher.com/antibody/product/Goat-anti-Rabbit-IgG-H-L-Highly-Cross-Adsorbed-Secondary-Antibody-Polyclonal/A-11034
56. Alexa-Fluor 568-conjugated goat anti-rabbit IgG, Thermo Fisher Scientific was validated by the supplier. https://www.thermofisher.com/antibody/product/Goat-anti-Rabbit-IgG-H-L-Highly-Cross-Adsorbed-Secondary-Antibody-Polyclonal/A-11036
57. Alexa-Fluor 488-conjugated goat anti-mouse IgG, Thermo Fisher Scientific was validated by the supplier. https://www.thermofisher.com/antibody/product/Goat-anti-Mouse-IgG-H-L-Highly-Cross-Adsorbed-Secondary-Antibody-Polyclonal/A-11029
58. Alexa-Fluor 568-conjugated goat anti-mouse IgG, Thermo Fisher Scientific was validated by the supplier. https://www.thermofisher.com/antibody/product/Goat-anti-Mouse-IgG-H-L-Highly-Cross-Adsorbed-Secondary-Antibody-Polyclonal/A-11031
59. Alexa-Fluor 633-conjugated goat anti-rabbit IgG, Thermo Fisher Scientific was validated by the supplier. https://www.thermofisher.com/antibody/product/Goat-anti-Rabbit-IgG-H-L-Highly-Cross-Adsorbed-Secondary-Antibody-Polyclonal/A-21245
60. Alexa-Fluor 633-conjugated goat anti-mouse IgG, Thermo Fisher Scientific was validated by the supplier. https://www.thermofisher.com/antibody/product/Goat-anti-Mouse-IgG-H-L-Highly-Cross-Adsorbed-Secondary-Antibody-Polyclonal/A-21236
61. HRP-conjugated goat anti-rabbit IgG, Jackson ImmunoResearch was validated by the supplier. https://www.jacksonimmuno.com/catalog/products/111-035-144
62. HRP-conjugated goat anti-mouse IgG, Jackson ImmunoResearch was validated by the supplier. https://www.jacksonimmuno.com/catalog/products/115-035-166

Eukaryotic cell lines

Policy information about cell lines and Sex and Gender in Research

Cell line source(s)	HeLa, HEK293T, and A549 (from ATCC), Vero E6 (from the NCCS Pune Cell Repository), and HEK293T-hACE2 (HEK293T cells expressing human angiotensin-converting enzyme 2 (ACE2); NR-52511; from BEI Resources) cells were cultured following the supplier's instructions. Stable cell lines (inducible or constitutive systems) were generated using the lentiviral transduction method (described under the “Cell Culture” section of Methods).
Authentication	All the cell lines were authenticated by the providers. HeLa, HEK293T, and A549 (from ATCC) and Vero E6 (from the NCCS Pune Cell Repository) were authenticated using STR profiling by the respective repositories. HEK293T-hACE2 (HEK293T cells expressing human angiotensin-converting enzyme 2 (ACE2); NR-52511; from BEI Resources) cells were authenticated using multiplex PCR amplification of the Cytochrome C Oxidase I (COI) gene for species identification, and expression of ACE2 was confirmed by indirect fluorescent antibody assay by the supplier. We also assessed the morphology of all the cell lines by microscopy. Each cell type was cultured for no more than 15 passages.
Mycoplasma contamination	All cell lines used in this study were regularly screened for the absence of mycoplasma contamination by using the MycoAlert. Mycoplasma Detection Kit (LT07-418, Lonza), and all the tests were negative for mycoplasma presence.
Commonly misidentified lines (See ICLAC register)	The study did not use any misidentified cell lines.

Plants

Seed stocks	N/A
Novel plant genotypes	N/A
Authentication	N/A

Flow Cytometry

Plots

Confirm that:

- [x] The axis labels state the marker and fluorochrome used (e.g. CD4-FITC).
- [x] The axis scales are clearly visible. Include numbers along axes only for bottom left plot of group (a 'group' is an analysis of identical markers).
- [ ] All plots are contour plots with outliers or pseudocolor plots.
- [x] A numerical value for number of cells or percentage (with statistics) is provided.

Methodology

Sample preparation	To determine the proteolytic activity of lysosomes, the cells were incubated for 2 h at 37°C in phenol red-free DMEM (Gibco)

containing 20 µg/mL BODIPY-FL-BSA (BioVision). After incubation, the medium was discarded and the cells were trypsinized, rinsed, resuspended in ice-cold 1X PBS, and then analyzed by flow cytometry (Kumar et al., 2022; Rawat et al., 2023). A BD FACS Aria Fusion Cytometer and BD FACS Diva software version 8.0.1 (BD Biosciences) were used to acquire the samples. Data analysis was performed using the BD FlowJo version 10.0.1.

Instrument	BD FACS Aria Fusion Cytometer was used in the study.
Software	FACS Diva software version 8.0.1 (BD Biosciences).
Cell population abundance	30,000 per sample were analysed.
Gating strategy	Viable cells were gated on a plot of FSC-A versus SSC-A.

☒ Tick this box to confirm that a figure exemplifying the gating strategy is provided in the Supplementary Information.
